# A Reproducible Bioprinted 3D Tumor Model Serves as a Preselection Tool for CAR T Cell Therapy Optimization

**DOI:** 10.3389/fimmu.2021.689697

**Published:** 2021-06-29

**Authors:** Laura Grunewald, Tobias Lam, Lena Andersch, Anika Klaus, Silke Schwiebert, Annika Winkler, Anton Gauert, Anja I. Heeren-Hagemann, Kathy Astrahantseff, Filippos Klironomos, Alexander Thomas, Hedwig E. Deubzer, Anton G. Henssen, Angelika Eggert, Johannes H. Schulte, Kathleen Anders, Lutz Kloke, Annette Künkele

**Affiliations:** ^1^ Charité – Universitätsmedizin Berlin, Corporate Member of Freie Universität Berlin and Humboldt-Universität zu Berlin, Department of Pediatric Oncology and Hematology, Berlin, Germany; ^2^ Freie Universität Berlin, Berlin, Germany; ^3^ Technische Universität Berlin, Berlin, Germany; ^4^ Cellbricks GmbH Berlin, Berlin, Germany; ^5^ Neuroblastoma Research Group, Experimental and Clinical Research Center (ECRC) of the Charité and the Max-Delbrück-Center for Molecular Medicine (MDC) in the Helmholtz Association, Berlin, Germany; ^6^ German Cancer Consortium (DKTK), Heidelberg, Germany; ^7^ German Cancer Research Center (DKFZ), Heidelberg, Germany; ^8^ Berlin Institute of Health at Charité – Universitätsmedizin Berlin, Berlin, Germany

**Keywords:** CAR T cells, neuroblastoma, T cell infiltration, 3D tumor model, bioprint technology

## Abstract

Chimeric antigen receptor (CAR) T cell performance against solid tumors in mouse models and clinical trials is often less effective than predicted by CAR construct selection in two-dimensional (2D) cocultures. Three-dimensional (3D) solid tumor architecture is likely to be crucial for CAR T cell efficacy. We used a three-dimensional (3D) bioprinting approach for large-scale generation of highly reproducible 3D human tumor models for the test case, neuroblastoma, and compared these to 2D cocultures for evaluation of CAR T cells targeting the L1 cell adhesion molecule, L1CAM. CAR T cells infiltrated the model, and both CAR T and tumor cells were viable for long-term experiments and could be isolated as single-cell suspensions for whole-cell assays quantifying CAR T cell activation, effector function and tumor cell cytotoxicity. L1CAM-specific CAR T cell activation by neuroblastoma cells was stronger in the 3D model than in 2D cocultures, but neuroblastoma cell lysis was lower. The bioprinted 3D neuroblastoma model is highly reproducible and allows detection and quantification of CAR T cell tumor infiltration, representing a superior *in vitro* analysis tool for preclinical CAR T cell characterization likely to better select CAR T cells for *in vivo* performance than 2D cocultures.

## Highlights

We present a highly reproducible bioprinted three-dimensional tumor model for preclinical *in vitro* CAR T cell evaluation allowing tumor and T cell characterization following experiments.

## Introduction

Genetically engineering a patient’s own primary T cells holds great immunotherapeutic promise. Chimeric antigen receptor (CAR) T cell therapy is currently receiving attention since treatments for various hematological malignancies in children and adults are showing remarkable clinical success (1, 2). Success has been limited for treating solid tumors with CAR T cell approaches, since CAR T cells need to find, enter and survive in a hostile tumor microenvironment (3), which require further improvement and preclinical testing. Here, we use CAR T cells targeting the glycosylated CE7 epitope of the L1 cell adhesion molecule, L1CAM (formerly CD171), which is specifically expressed on tumor cells and a promising target for neuroblastoma and ovarian carcinoma (4–6). Neuroblastoma is the most common extracranial solid tumor in childhood, and remains the third leading cause of pediatric cancer death despite multimodal therapies (7). Recently, children suffering from refractory neuroblastoma were treated with L1CAM-targeting CAR T cells in a clinical phase I trial (NCT02311621, https:clinicaltrials.gov) (4, 6).

The CAR construct expressed in T cells provides an extracellular single-chain variable fragment (scFv) derived from an antibody for specific antigen recognition, fused to a variably long spacer domain and transmembrane domain. The transmembrane domain connects to the CD3 zeta (ζ) signaling domain of the T cell receptor (1^st^ generation) and is embellished with one (2^nd^ generation) or two (3^rd^ generation) intracellular costimulatory domains. The most commonly used costimulatory domains are 4-1BB and CD28. Costimulus with 4-1BB is associated with a slower, more continuous anti-tumor response comparable to a memory T cell response, whereas a strong fast, effector-like T cell response is induced by CD28 costimulation (8). Minor differences in CAR design, such as spacer length, can significantly impact CAR T cell functionality (9, 10). Excessive *in vitro* testing followed by labor-intensive and time-consuming preclinical evaluation in mouse models are currently necessary to select the most suitable CAR construct for a given antigen.

CAR T cell effector functions are currently most often evaluated in two-dimensional (2D) cocultures where T cells encounter a tumor cell monolayer growing adherently on culture plastic. Three-dimensional (3D) connections to cells and matrix components in the tumor environment can influence cancer cell phenotype, including gene expression, cell signaling and nutrient supply (11). These influences are lacking in a cancer cell monolayer, in which tumor cells are also easily accessible to T cells, poorly reflecting cellular and matrix obstructions that T cells face in the *in vivo* tumor environment. CAR T cell efficacy achieved in cocultures often cannot be achieved in preclinical mouse models, extending the animal testing necessary to select CAR T cell candidates. The dramatic evolution of 3D printing technology over the past decades [reviewed in (12) and (13)] enables an innovative approach to *in vitro* testing, surpassing the environment created in 2D cocultures. Stereolithography combines high resolution and speed with the ability to simultaneously print large numbers of objects with high reproducibility (14). This technology uses photopolymerization to sequentially solidify layers of bio-ink printed on top of each other to build a 3D structure mimicking native tissues and demonstrating significant improvements to 2D cocultures (15).

Our aim was to build on data from our previous comparisons of a L1CAM-specific CAR T cell constructs harboring either long or short spacers and the 4-1BB costimulatory domain, which showed functional discrepancies between preclinical evaluations *in vitro* and in mouse models, and are used in the ongoing clinical phase I trial for children with neuroblastoma (ClinicalTrials.gov Identifier: NCT02311621) (6). Here we repeated the functional evaluation of L1CAM-specific CAR T cells with different spacer lengths in 2D cocultures and directly compared functionality with that in bioprinted 3D neuroblastoma models.

## Materials and Methods

### Neuroblastoma Cell Culture

SK-N-BE(2) neuroblastoma cells (passaged ≤20 times from stock cultures expanded in <10 passages from the source culture obtained from ATCC) were propagated in Dulbecco´s Modified Eagle Medium (Life Technologies, Karlsbad, CA, USA) supplemented with 10% heat-inactivated fetal calf serum (Sigma-Aldrich, St. Louis, MO, USA) to 80% density in 2D culture before 3D bioprinting or seeding for functional assays. The identity of the SK-N-BE(2) neuroblastoma cell line was confirmed by Eurofins (Luxemburg) and *Mycoplasma*-negative by a cell-based colometric HEK-Blue Detection assay (Invivogen).

### Bioprinting 3D Tumor Models

Stereolithographic bioprinting using a previously described process (16) was selected to produce the 3D models. Methacrylated gelatin (GelMA) was synthesized for use in the bio-ink as previously described (17, 18) with slight changes. In short, 10 weight percent (wt%) type A gelatin from porcine skin (300 bloom, Sigma-Aldrich) was dissolved in phosphate buffered saline (PBS) at 50°C before adding methacrylic anhydride (Sigma-Aldrich) in molar excess, and allowed the reaction to proceed 2h. Resulting GelMA was dialyzed against distilled water for 4 days with frequent water changes, then sterile filtered and lyophilized several days until dry. NMR spectroscopy determined 60% methacrylation in the lyophilized product. Bio-ink was prepared using 7wt% GelMA in cell culture medium supplemented with 10% fetal calf serum and 1% penicillin/streptomycin, with 0.1wt% lithium phenyl-2,4,6-trimethylbenzoy phosphinate as photoinitiator. SK-N-BE(2) cells were harvested, counted and viability was controlled, before resuspending in the GelMA mixture at 10^8^ cells/mL to create the bio-ink. A computer-assisted design model (CAD) was created using Rhinoceros 5 software (McNeel Europe, Barcelona, Spain) for the final 3D model architecture with a diameter of 4 mm and a height of 500 µm, then computationally sliced for processing by the bioprinter. Each layer of bio-ink was printed onto the print head then photopolymerized (cured) using blue light in the wavelength range of 385 – 405nm as previously described (16). The individual 3D tumor models were printed in a layer-by-layer fashion therefore achieving a homogeneous distribution of cells throughout the 3D tumor model. After printing, the models were washed in PBS to remove excess liquid bio-ink and cultivated in cell culture medium in a multi-well plate at 37°C and 5% CO_2_ atmosphere.

### Assaying Viability in Bioprinted 3D Tumor Models

Cell viability was determined using an NC-200™ NucleoCounter^®^ (Chemometec). Prior to their suspension in the bio-ink for printing, neuroblastoma cells were stained with CellTracker™ Red CMTPX dye (Thermo Fisher Scientific, Waltham, MA, USA) according to the manufacturer’s protocol with prolonged incubation times, then washed in PBS and counter-stained with CellTox™ Green dye (Promega, Madison, WI, USA) according to the manufacturer’s protocol. The stained 3D tumor models were analyzed using a 2-photon microscope (LaVision Biotec GmbH, Bielefeld, Germany). A region of interest comprising 2.5 x 2.5 x 0.3mm from 5 models was imaged two and eleven days after printing. Imaris Software 7.6.5 (Oxford Instruments, Oxford, UK) was used to count red cells (total cell number) and green cell cores (dead cells) were counted. Live/dead cell ratios in percent were calculated using the simple formula:

$$Cell\;viability\;[\%]=\frac{total\;cell\;count-green\;cell\;count}{total\;cell\;count}*100$$

### CAR Construct and CAR T Cell Generation

The previously described L1CAM-specific CE7-CAR (19) was cloned into the SIN epHIV7 lentiviral vector then propagated in 293T cells and isolated as previously described (20). The single-chain variable Fragment (scFv) in the CAR construct was codon optimized and subsequently linked to a 12 (short) or 229 (long) amino acid spacer domain from the human IgG4-Fc hinge. The long spacer domain was modified by substituting L235D and N297Q to reduce binding to the IgG Fc gamma receptor (on natural killer cells and monocytes), which causes unintended CAR T cell activation *via* innate immune cell activation (21). The spacer domain connects the antigen-binding domain to CD28 transmembrane domain followed by the signaling module containing the CD3zeta (ζ) cytoplasmic domain and the 4-1BB (second generation CAR) costimulatory domain. CAR constructs were linked downstream to a T2A self-cleaving peptide and a truncated epidermal growth factor receptor (EGFRt) allowing CAR T cell detection and enrichment (22). CAR T cells were generated from healthy donors (Charité ethics committee approval EA2/216/18) as previously described (19). T cells used as controls alongside CAR T cells in experiments were not lentivirally transduced. CAR and control T cells were cryopreserved until further use. Cryopreserved cells were thawed and stimulated with irradiated peripheral blood mononuclear cells, irradiated CD19^+^ EBV-transformed lymphoblastoid cell line (TM-LCL), and 30ng/mL antibody activating the CD3 complex (OKT3 clone, Miltenyi Biotec, Bergisch Gladbach, Germany). For rapid expansion, T cells were maintained in RPMI 1640 media supplemented with 10% fetal calf serum, 0.5ng/mL IL15 (Miltenyi Biotec) and 50U/mL IL2 (Novartis, Basel, Switzerland) according to a rapid expansion protocol (23). Functional *in vitro* assays were conducted between days 11 and 16 of culture adding fresh IL2 and IL15 to the coculture experiments.

### Immunofluorescent Marker Detection in Bioprinted 3D Tumor Models

To visualize cells in their orientations in the bioprinted 3D model, models were fixed in 4% paraformaldehyde, embedded in paraffin and sectioned into 5µm slices using a microtome (HM 340E, Thermo Fisher Scientific). Immunofluorescence staining was performed with fluorochrome-conjugated antibodies diluted in blocking buffer directed against mouse monoclonal anti-human L1CAM (clone UJ127.11; Thermo Fisher Scientific) 1:500, rabbit monoclonal anti-human CD3 (clone SP7; Abcam, Cambridge, UK) 1:100 on sections overnight at 4°C. Recommended secondary antibodies were diluted in 1:500 and incubated for 1 hour. Nuclei were counterstained with Hoechst (B2261 Sigma Aldrich) diluted in 1:5,000-1:10,000 stain. Another staining approach for the bioprinted 3D tumor model was conducted by prestaining tumor cells with CellTracker™ Red CMTPX dye (Thermo Fisher Scientific) prior bioprinting. T cells were prestained with CellTracker™ Green CMFDA dye (Thermo Fisher Scientific) before coculture and following live-cell imaging. Images were acquired using a Nikon eclipse Ti-A1 microscope (Nikon, Tokyo, Japan) or a Leica M165 FC (LAS X software, Leica, Wetzlar, Germany) microscope. Cross sections of immunofluorescently stained samples were analyzed using Fiji ImageJ and MorpholibJ software. T cell infiltration was assessed from vertical scans conducted at 3 arbitrary locations in each bioprinted 3D model. Vertical scans were conducted in 1µm partitions in which T cells were identified by the mean red fluorescence, and mean fluorescence intensity was normalized to between 0 and 1 for combination of the vertical partitions and calculation of the depth of overall T cell infiltration (µm from the model surface).

### Enzymatic Digestion of Bioprinted 3D Tumor Models

Bioprinted 3D tumor models could be processed into a single-cell suspension by digestion of non-cellular material using an enzymatic cocktail of 0.1% dispase II, 0.01% DNase I, 0.01% papain and 12.4 mM MgSO_4_ in Hank´s balanced salt solution as described (24). Briefly, individual bioprinted 3D tumor models were washed with PBS and then each mechanically minced into small pieces using a scalpel in a culture dish. Minced pieces were digested in the enzymatic cocktail for 20-30 minutes at room temperature directly in the culture dish. To assist matrix dissolution and release of single cells, tumor model pieces were gently triturated through a 1000 µL pipette tip every 5 minutes. The resulting cell suspension was centrifuged at 300 x g for 5 minutes, before discarding supernatant and washing twice in PBS.

### Flow Cytometric Marker and Antigen Detection

Cell surface expression of L1CAM (clone REA163, Miltenyi Biotec), GD2 (clone 14.G2a; BD), CD3 (clone Hit3a, BioLegend, San Diego, CA, USA) and CD8 (clone SK1; BioLegend) was detected by fluorophore-conjugated monoclonal antibodies on a Fortessa X-20 (BD Biosciences, Franklin Lakes, NJ, USA) 4-laser flow cytometer. EGFRt expression was detected using biotinylated cetuximab (Bristol-Myers Squibb, New York, NY, USA) and a phycoerythrin (PE)-conjugated streptavidin antibody (cat #12-4317-87, BioLegend). Activation was assessed by fluorophore-conjugated monoclonal antibodies detecting TNFRSF9 (formerly CD137, clone 4B4-1; BioLegend) and IL2RA (formerly CD25, clone BC96; BioLegend). The Annexin V/7-AAD detection kit (BioLegend) was used to assess apoptosis. Dead cells were excluded from analyses using the LIVE/DEAD™ Fixable Green Dead Cell Stain Kit (cat#L23101, Life Technologies). Precision count beads (BioLegend) were used to quantify T cell infiltration according to the manufacturer’s instructions. Flow cytometry data was processed using FlowJo_V10 Software (Tree Star Inc., Ashland, OR, USA).

### Cytokine Release Assays

For cytokine release assays, 3x10^6^ T cells were seeded into wells (24-well plates) together with stimulator cells at a 5:1 effector:target ratio. All data points were performed as technical triplicates. After 12, 24, 36, 72 and 120 hours, supernatants were collected and stored at -80°C until analysis of IFNG using the OptEIA™ Set (BD Biosciences) ELISA kits in accordance with the manufacturer’s instructions.

### Statistical Analysis

Differences in cytotoxic activity, cell surface marker expression and cytokine release between treatment groups and controls were analyzed using the paired or unpaired Student’s T test in GraphPad prism 8 software (GraphPad Software, La Jolla, CA, USA). All experiments were independently repeated (n = 3 or 4). P values <0.05 were considered statistically significant.

## Results

### Neuroblastoma Cells Can Be Stereolithographically Bioprinted Into a 3D Tumor Model

**Figure 1:**
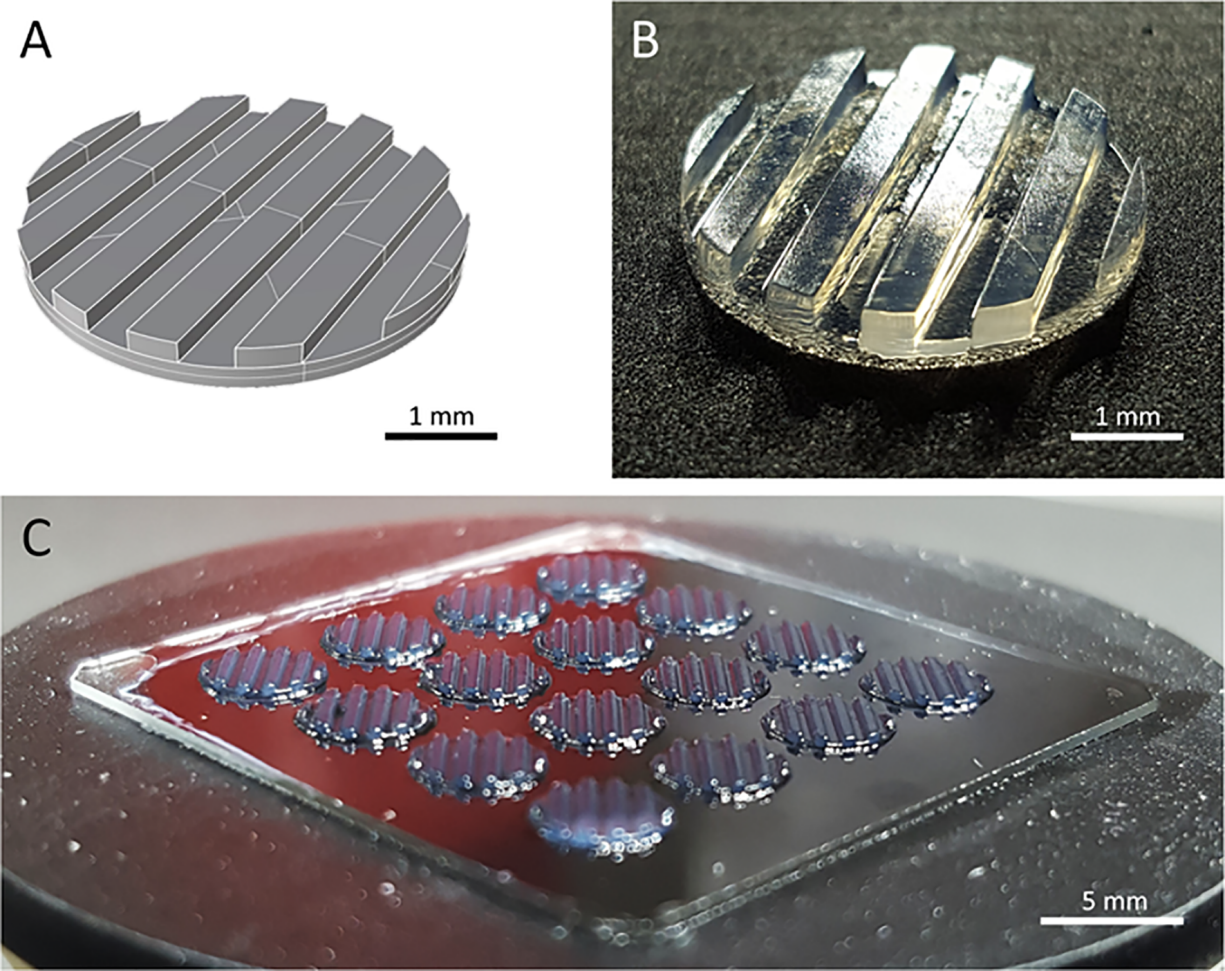
The bioprinted 3D neuroblastoma tumor model. **(A)** Computer-assisted design file (CAD) used for printing the 4 mm diameter and 500 µm depth model. **(B)** Macroscopic photograph of bioprinted 3D tumor model. Scale bar = 1 mm. **(C)** An array of 16 multiple tumor models directly after printing. Scale bar = 5 mm.

Two major challenges for CAR T cell therapies used against solid tumors, are tumor infiltration and preservation of functionality upon massive antigen encounter (25, 26). Currently used 2D *in vitro* models cannot address these challenges. We developed a 3D tumor model that allows analysis of CAR T cell infiltration and phenotype. A digital, computer-assisted design (CAD) file was generated for the desired design. The 3D tumor model design used a disk with channel-like features that increased the surface area for CAR T cell interaction with tumor components (**Figure 1A**). Since channel-like features were located along the top surface (open to culture medium) of the bioprinted 3D tumor model, this structured surface also preserved orientation of the 3D model during cultivation, manipulation and post-experiment analyses including the preparation of slices for microscopic visualization. The physical 3D tumor model was then created by stereolithographically bioprinting a bio-ink composed of the established human SK-N-BE(2) neuroblastoma cell line suspended in 7% methacrylated gelatin with a photoinitiator (**Figure 1B**). Methacrylated gelatin forms an extracellular matrix allowing the cell migration necessary to study CAR T cell invasion in the models. As our technology is not dependent on extruders, but only on the size of the build space, 3D models can be produced in parallel in one production step. Our light projection-based bioprinting technology enabled the simultaneous printing of 16 neuroblastoma tumor models in parallel in this study, creating a high degree of comparability within each experiment (**Figure 1C**). These data demonstrate that stereolithographic bioprinting can be used to produce multiple bioprinted 3D neuroblastoma models at a time.

### Bioprinted 3D Neuroblastoma Models Remain Viable Over Time

**Figure 2:**
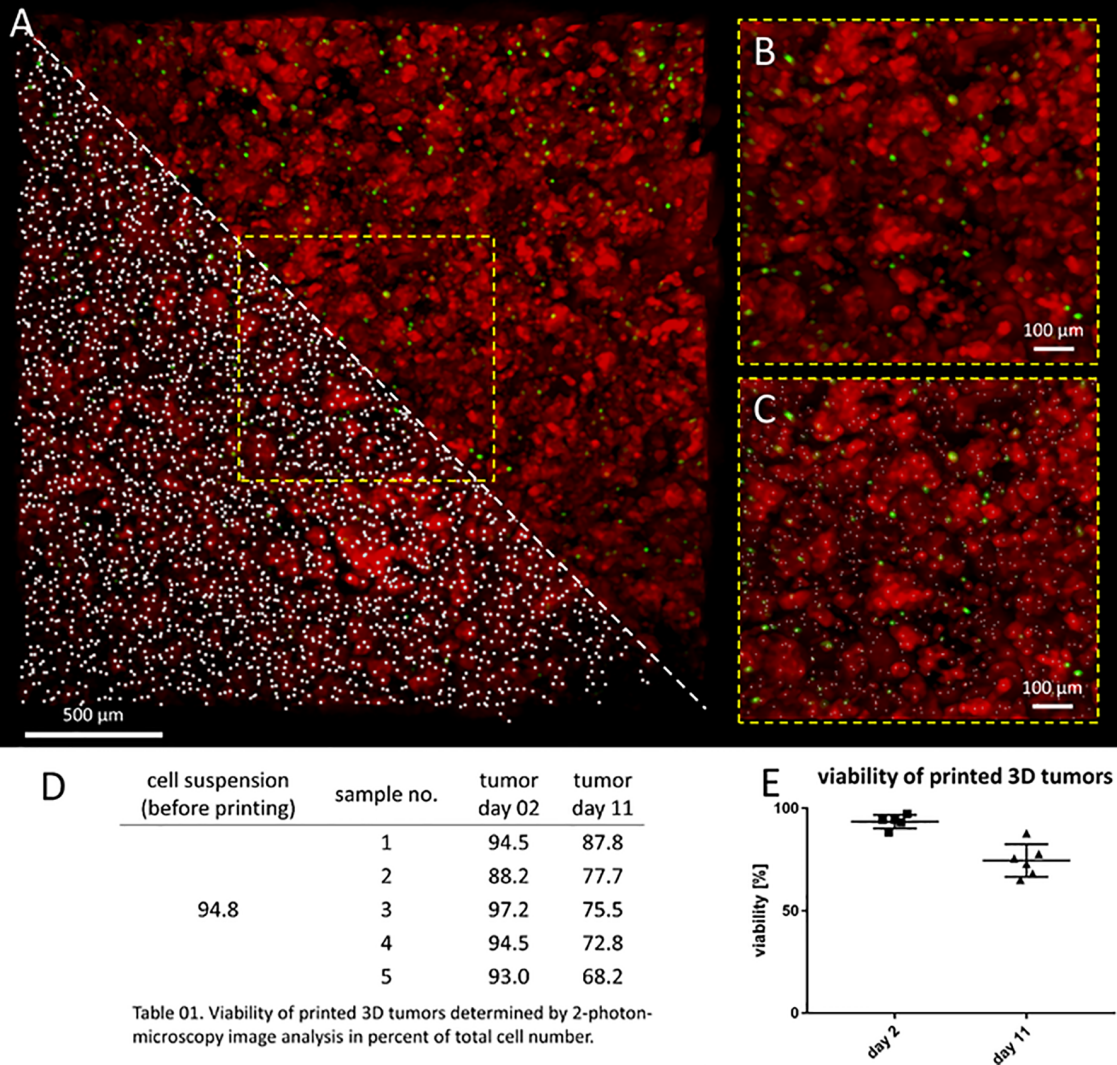
Neuroblastoma cells remain viable over 11 days in bioprinted 3D tumor model. **(A)** Image shows 0.75mm³ cut-out of bioprinted 3D tumor model, which was visualized by 2-photon image analysis. Viable cells are stained by CellTracker^TM^ red and dead cells by CellTox^TM^ green. A region of interest is enlarged without marking cells (upper triangle and **B**) and by computationally counting cells visualized by white dots (lower triangle and **C**) Scale bar = 100 µm. Neuroblastoma cell viability both before and after printing is presented in the table **(D)** and graph **(E)**. Viability of printed cells after 2 days and 11 days in culture was determined by 2-photon image analysis.

Model usefulness for preclinical testing requires that tumor cells living in the 3D models retain their viability for several days. Most experiments to test CAR T cell efficacy and activity require 3-5 days, but we purposely chose to assess an extreme experimental window to explore maximal experimental support with this novel 3D tumor model. We assessed the viability of neuroblastoma cells in the bio-ink before printing and the bioprinted 3D models after 2 and 11 days of cultivation using 2-photon image analysis. SK-N-BE(2) cells were labeled with CellTracker™ and CellTox™ before printing, to distinguish viable (red) from dead (green) cells in the 2-photon image analysis (**Figures 2A–C**). Cell viability in the bio-ink suspension was 94.8%. Viability in cell suspensions isolated from 5 bioprinted 3D models averaged 93.0% (range 88.2-97.2%) two days after printing and 76.0% (range 68.2-87.8%) eleven days after printing (**Figures 2D, E**). These data demonstrate that on average 76.0% of neuroblastoma cells in the model remain viable even after 11 days of cultivation in the bioprinted 3D model, supporting preclinical analyses of CAR T cell infiltration and T cell status even in extended experimental designs testing multiple antigen encounters.

### CAR T Cells Can Infiltrate the Bioprinted 3D Tumor Model

**Figure 3:**
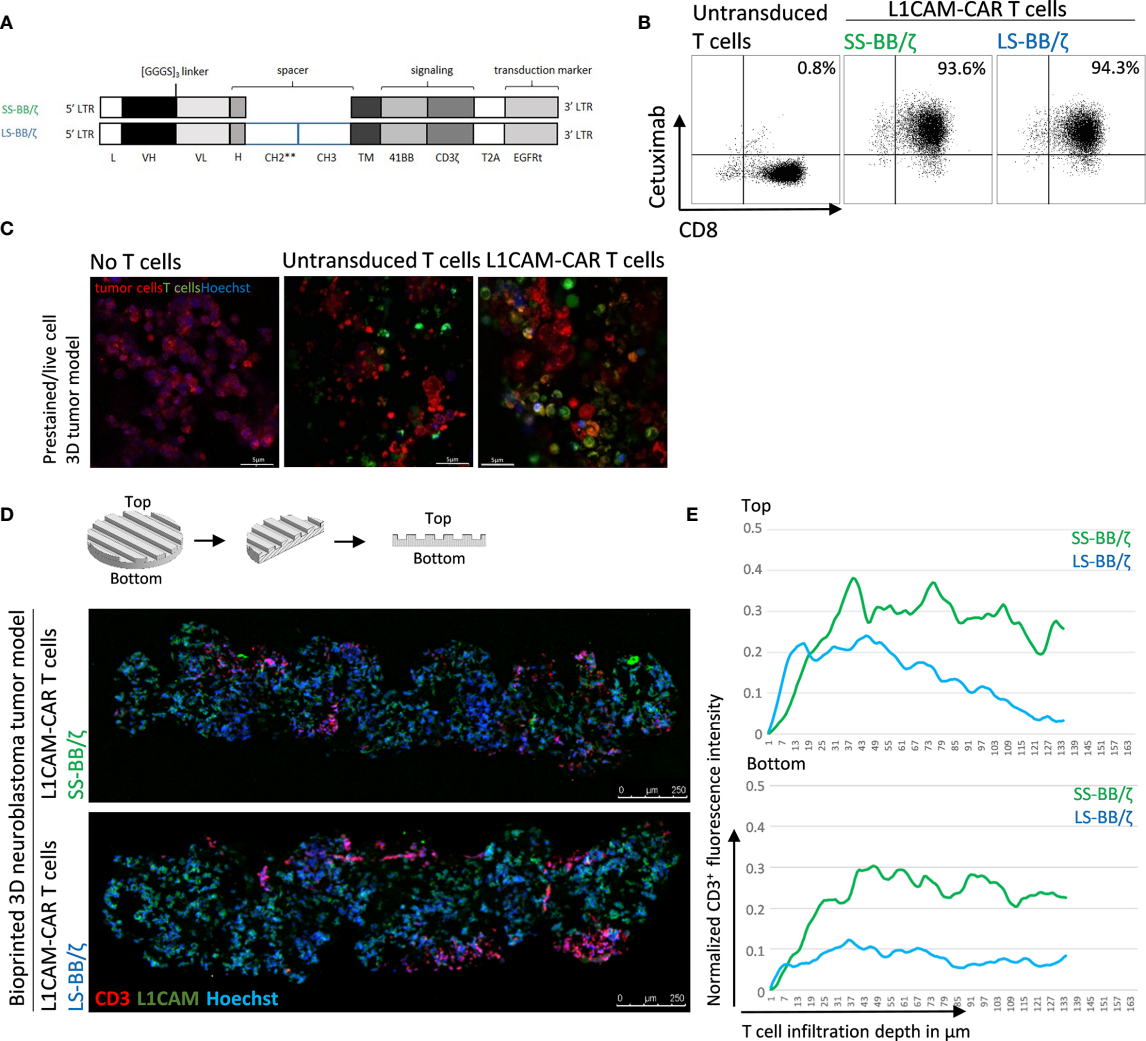
L1CAM-specific CAR T cells in coculture with bioprinted 3D neuroblastoma tumor model. **(A)** Schematic representation of lentiviral constructs used to generate L1CAM-specific second-generation CAR T cells. L, long-terminal repeat; VH, variable region of the heavy chain; VL, variable region of the light chain; H, hinge region with either short (IgG4) or long (CH2**-CH3) spacer; CH2**, CH2 harboring both L235D and N297Q point mutations; TM, transmembrane domain; T2A, virus 2A self-cleaving sequence. **(B)** Representative flow cytometry plots showing EGFRt transduction marker expression on CD8+ T cells transduced with L1CAM-specific short spacer 4-1BB zeta (SS-BB/ζ) and long spacer 4-1BB zeta (LS-BB/ζ) constructs after enrichment. Untransduced T cells served as negative control. **(C)** Live-cell imaging of prestained bioprinted 3D tumor models (lower panel) alone (prestained with CellTracker™ Red CMTPX, red and Hoechst, blue) or in coculture with untransduced or LS-BB/ζ T cells (prestained with CellTracker™ Green CMFDA, green, and Hoechst, blue) using confocal microscopy (E:T = 5:1) Scale bar = 5 µm. **(D)** Schematic depiction of 3D print and FFPE sample processing and orientation. Immunofluorescence staining of formalin-fixed paraffin-embedded (FFPE) 3D tumor models treated with L1CAM-CAR T cells for CD3 (red), L1CAM (green) and Hoechst (blue). Schematic 3D print serves for orientation; Scale bar = 250 µm. **(E)** T cell infiltration depth quantified by red fluorescence channel profiling. Staining intensity is depicted as normalization of CD3+ T cells fluorescence intensity on the y-axis, and T cell infiltration depth into the bioprinted 3D tumor is represented on the x-axis. Top indicates the upper model surface containing the channels. Bottom indicates the flat lower model surface. Depicted is the mean of three distinct areas from biological triplicates.

Conventional coculture of CAR T cells with an adherent tumor cell monolayer has been extensively used to preclinically evaluate CAR T cell efficacy, mainly utilizing cytokine production, tumor cell lysis and T cell exhaustion as endpoints in short-time experiments (27, 28). These models, however, do not support assessment of the ability of CAR T cells to infiltrate the solid tumor structure, an ability necessary for tumor eradication. Here, we test whether CD8^+^ L1CAM-specific CAR T cell infiltration can be assessed using a bioprinted 3D neuroblastoma model, in which the embedded neuroblastoma cells express the target antigen, L1CAM. We engineered T cells to express a second-generation L1CAM-specific CAR with 4-1BB as costimulatory domain harboring either a short (SS-BB/ζ) or a long (LS-BB/ζ) spacer (**Figure 3A**). Following immunomagnetic selection for EGFRt, >90% of T cells expressed either CAR construct used (**Figure 3B**). Untransduced T cells served as a negative control. We microscopically investigated whether we see L1CAM-CAR T cells within the bioprinted 3D tumor models. For detailed analysis, live-cell confocal microscopy was used to analyze prestained CAR T cells (green) in coculture with prestained neuroblastoma cells (red) in the bioprinted 3D tumor model. Live-cell imaging revealed T cell proximity to tumor cells and infiltration of untransduced and L1CAM-CAR T cells into the bioprinted 3D models (**Figure 3C**). We also applied immunofluorescence staining to determine whether CAR T cells infiltrated the bioprinted 3D tumor model. Samples were formalin-fixed and paraffin-embedded (FFPE) 24 hours after introducing CAR T cells to the bioprinted 3D model, then sectioned before detecting L1CAM and CD3 with Hoechst counterstaining to detect nuclei (**Figure 3D**). T cell infiltration into the bioprinted 3D tumor model was determined by analyzing three distinct areas of three individually stained T cell-treated 3D tumor models (**Supplementary Figure 1A**). CAR T cell infiltration depth was investigated by normalizing CD3^+^ (red) immunofluorescence (**Figure 3E**). Interestingly, both L1CAM-specific CAR T cell subsets infiltrated into the top (indicated by the peaks between channels) and bottom of the bioprinted 3D tumor models. A region within the sections from the bioprinted 3D tumor model was viewed under higher magnification to observe T cell proximity to tumor cells (**Supplementary Figure 1B**). These results demonstrate that our bioprinted 3D neuroblastoma model is suitable for visualizing and quantifying target-specific CAR T cell infiltration either by live-cell imaging using prelabeled (Celltracker™) cell populations or using immunofluorescence staining of cryopreserved or FFPE specimens.

### Single-Cell Suspensions Can Be Harvested From Bioprinted 3D Models for Functional Assays

**Figure 4:**
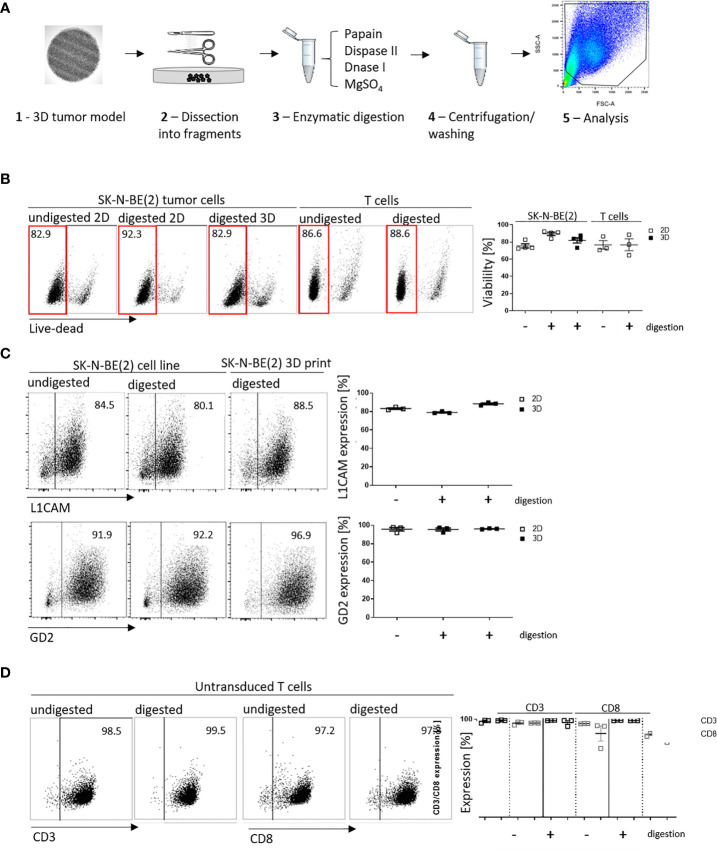
Cell viability and cell surface molecule expression is maintained after enzymatic digestion. **(A)** Schematic depiction of enzymatic digestion protocol to produce single-cell suspensions after 3D model experiments. **(B)** Representative flow cytometry plots (left) showing live (framed in red) and dead cells that either underwent the enzymatic digestion protocol or not (from either 2D or suspension cultures) and the single-cell suspension from the 3D models. Numbers at the top of each plot indicate percent of the total cell population. SK-N-BE (2) neuroblastoma and untransduced T cells are shown in separate panels of the representative flow cytometry plots. Scatter plots on the right summarize results from 3 or 4 individual experiments. **(C)** L1CAM and GD2 antigen expression analyzed by flow cytometry (representative plots shown, left), and is summarized from 3 experiments in the scatter plot (right). **(D)** Representative CD3^+^ and CD8^+^ surface molecule expression on untransduced T cells without and with enzymatic digestion are shown (left) and summarized in the scatter plot (n = 3, right). All experiments were conducted after 24h of coculture.

We have shown that infiltrated L1CAM-CAR T cells can be visualized with different microscopic approaches. Next, we tested whether CAR T cell-treated bioprinted 3D tumor models could be processed into a single-cell suspension allowing flow cytometric analysis of both tumor and CAR T cells from treatment groups. This would present a highly useful protocol for experimental analyses or endpoint measurements requiring single cells or multiple cells freed from their interacting components and enhance bioprinted 3D tumor model experimental applications. Different mechanical (not shown) and enzymatic treatment methods for dissociation were tested. The method of choice was to digest non-cellular material in the bioprinted 3D models using an enzymatic cocktail (see *Materials and Methods* section; **Figure 4A**). To check if enzymatic digestion reduced cell viability, 2D tumor and T cell monocultures were digested with the same enzymatic cocktail alongside the bioprinted 3D models, then cell viability was flow cytometrically determined using a fluorescent dye labeling dead cells. The digested 3D neuroblastoma models, as well as SK-N-BE(2) and L1CAM-CAR T cells enzymatically digested from 2D monocultures were as viable as their undigested controls (**Figure 4B**). Maintenance of protein expression during sample preparation is highly relevant for immunological research. We flow cytometrically analyzed expression of the L1CAM and GD2 surface antigens on neuroblastoma cells as well as CD3^+^ and CD8^+^ surface molecules on L1CAM-specific CAR T cells and untransduced T cells after enzymatic digestion. L1CAM expression on SK-N-BE(2) cells digested from 2D or bioprinted 3D models did not differ from undigested SK-N-BE(2) cells (digested 2D: 79.0 ± 1.1%, digested 3D: 88.3 ± 1.7%, undigested 2D: 83.2 ± 1.5%; **Figure 4C**). Similar results were obtained for GD2 expression (digested 2D: 95.4 ± 2.8%, digested 3D: 96.1 ± 0.5%, undigested 2D: 95.7 ± 3.3%; **Figure 4C**). Enzymatic digestion also did not remove CD3^+^ and CD8^+^ surface molecules from L1CAM-specific CAR T cells (**Supplementary Figure 2A**) or untransduced T cells (CD3^+^: digested: 98.8 ± 0.4%, undigested: 97.9 ± 1.6%; CD8^+^: digested: 96.2 ± 0.1%, undigested: 96.0 ± 1.7%; **Figure 4D**). These data demonstrate that bioprinted 3D tumor models and target-specific CAR T cells maintain their cell viability and, importantly, their surface molecule expression during sample preparation for single-cell suspensions following experiments conducted in the bioprinted 3D models, enhancing bioprinted 3D tumor model usefulness for CAR T testing and experimentation.

### CAR T Cell Activation Is Superior in the Bioprinted 3D Neuroblastoma Model

**Figure 5:**
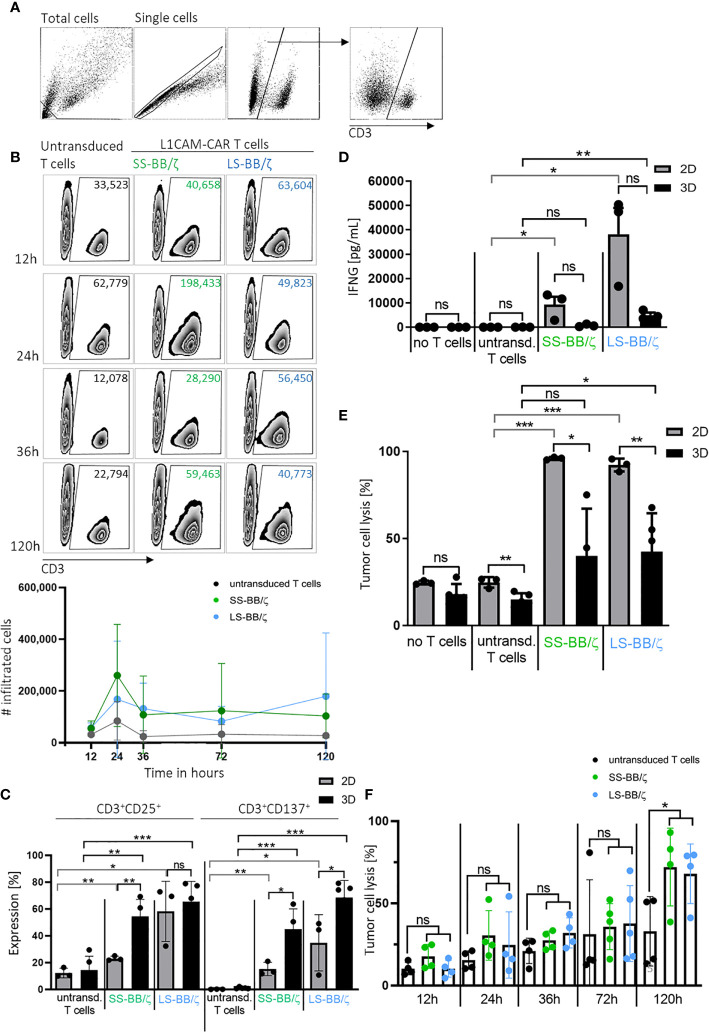
L1CAM-CAR T cells infiltrate and are highly activated in bioprinted 3D neuroblastoma tumor models. **(A)** Gating strategy for flow cytometry (applied with FlowJo_V10) is shown. Gates are applied to distinguish single viable T (CD3^+^) and tumor cells (CD3^-^) within the total cells. **(B)** Representative zebra plots showing numbers of CD3^+^ viable cells that infiltrated the 3D tumor model after the indicated time measured by flow cytometry with precision counting beads including summary of 3 biological replicates. **(C)** Surface activation markers were flow cytometrically analyzed on untransduced T cells and L1CAM-specific CAR T cells after 24h of tumor cell interaction (effector:target ratio of 5:1) in either 2D coculture or the bioprinted 3D model. Depicted are double-positive cells for CD8^+^ and CD25 or CD137. Cells were gated from living single cells. Bars depict the mean of 3 (2D) or 4 (3D) experiments with error bars representing SD. **(D)** Interferon gamma (IFNG) released into the culture media was detected by ELISA after 24h of tumor cell interaction (effector:target ratio of 5:1) in either 2D coculture or the bioprinted 3D model. Bars depict the mean of 3 (2D) or 4 (3D) experiments with error bars representing SD. **(E)** Tumor cell cytotoxicity was analyzed (FACS) after 24h of tumor cell interaction (effector:target ratio of 5:1) in either 2D coculture or the bioprinted 3D model. Bars depict the mean of 3 (2D) or 4 (3D) experiments with error bars representing SD. **(F)** FACS-based tumor cell cytotoxicity is shown for the 5-day time course in the bioprinted 3D model after addition of untransduced T cell controls or L1CAM-specific CAR T cells, as indicated. Experiments tested L1CAM-specific CAR T cells with either the short (green) or long (blue) spacer. Bars depict the mean of 4 experiments with error bars representing SD. ns, not significant, *p ≤ 0.5, **p ≤ 0.01, ***p ≤ 0.001.

An important prerequisite for effective therapy is CAR T cell infiltration into tumor tissue. After confirming that T cell-treated bioprinted 3D tumor models can be enzymatically dissociated into single-cell suspensions without reducing expression of key T cell surface markers, we used CD3^+^ cell surface staining and counting beads to flow cytometrically quantify CAR T cell infiltration. A kinetic profile of L1CAM-specific CAR T cell infiltration into the bioprinted 3D tumor model was constructed by detecting CD3^+^ cell populations in 3D models at 12, 24, 36 and 120 hours using a flow cytometry gating strategy (**Figure 5A**) on the dissociated single-cell suspensions to quantify CD3^+^ populations in the 3D model over time in zebra plots (**Figure 5B**). After only 12 hours, 63,604 LS-BB/ζ L1CAM-CAR T cells, 40,658 SS-BB/ζ L1CAM-CAR T cells and 33,523 untransduced T cells had infiltrated the 3D tumor model. Highest CAR T cell infiltration was detected after 24 hours, when 198,433 SS-BB/ζ L1CAM-CAR T cells and 49,823 LS-BB/ζ L1CAM-CAR T cells had infiltrated the tumor model (**Figure 5B**). Antigen-independent infiltration of untransduced T cells remained relatively stable over the testing period (**Figure 5B**). We assessed T cell activation markers at the time of peak infiltration (24h). The proportion of T cells expressing both CD25 and CD137 activation markers was higher in bioprinted 3D tumor models, where we solely measured activation of total infiltrated T cells, compared with 2D monolayer cocultures, where all T cells were included (**Figure 5C**). CD137 and CD25 expression was higher on L1CAM-CAR T cells with the long spacer compared to their counterparts using the short spacer in assays using either 2D or 3D models. Untransduced T cells expressed minimal CD25 and no CD137, excluding unspecific T cell activation. CAR T cell activation in 2D or 3D cocultured cells was significantly higher compared to paired untransduced T cells. Tumor cell encounter in the bioprinted 3D tumor model induced release of lower levels of the cytokine, interferon gamma (IFNG), from L1CAM-CAR T cells than encounter in 2D cocultures (SS-BB/ζ: p=0.07; LS-BB/ζ: p=0.06; **Figure 5D**). CAR T cell-mediated tumor cell cytotoxicity was analyzed using a fluorescent dye labeling dead cells and a gating strategy identifying the CD3^-^ tumor cells at the 24-hour time point (**Supplementary Figure 2B**). L1CAM-specific CAR T cells more easily killed tumor cells in 2D cocultures (SS-BB/ζ L1CAM-CAR T cells lysed: 95.9 ± 0.8% in 2D, 39.9 ± 27.1% in 3D; LS-BB/ζ L1CAM-CAR T cells lysed: 92.1 ± 3.6% in 2D and 42.4 ± 22.0% in 3D; **Figure 5E**). Control experiments induced only low-level tumor cell lysis in both 2D (no T cells: 24.6 ± 1.0%, untransduced T cells: 24.7 ± 3.0%) and 3D (no T cells: 18.0 ± 5.8%, untransduced T cells: 14.8 ± 3.6%) models (**Figure 5E**). The kinetics of neuroblastoma cytotoxicity in the bioprinted 3D tumor model was analyzed at 12, 24, 36, 72 and 120 hours, with maximal cytotoxicity being reached only after 120 hours of CAR T cell treatment and lysing 72.0 ± 23.6% (SS-BB/ζ) and 67.9 ± 18.1% (LS-BB/ζ) of neuroblastoma cells, which is significantly higher compared to untransduced T cells (**Figure 5F**). L1CAM-specific CAR T cells were more strongly activated in the bioprinted 3D tumor model, but induced less IFNG release than in 2D cocultures. The bioprinted 3D tumor model supported the detection and quantification of T cell infiltration into the tumor model, expanding *in vitro* testing possibilities.

## Discussion

Current strategies to analyze CAR T cell effector function heavily rely on *in vitro* analyses in 2D culture models, which only limitedly represent solid tumor physiology. CAR T cells must home to the tumor site, circumvent inhibitory effects of the tumor microenvironment and persist during multiple rounds of antigen encounter to eradicate solid tumors (25, 26). Widely used 2D coculture systems cannot investigate these hurdles. We developed a novel method to analyze CAR T cell effector function in a stereolithographically bioprinted 3D tumor model, and present proof-of-concept here using CAR T cells targeting one neuroblastoma target protein, L1CAM, and a neuroblastoma cell line. Our model is easily extensible to other tumor types and CAR T cell targets. We present live-cell and fluorescence microscopy methods to visualize interactions between second-generation L1CAM-specific CAR T cells with the 4-1BB costimulatory domain harboring the short or long spacer and tumor cells in these bioprinted 3D neuroblastoma models. We also present an optimized protocol to convert a bioprinted 3D tumor model experiment into a single-cell suspension, maintaining cell viability and surface protein markers descriptive of the cell phenotypes. This method supports analysis not only of T cell infiltration but CAR T cell activation and effector function for advanced quantitative endpoint analysis in 3D tumor model experiments.

Tumor cells clustered into spheroids from a single cell line or heterogeneous tumor cell types have been used to assess antibody (29), natural killer cell (29–31), cytotoxic T cell (32) and CAR T cell (33–35) immunotherapy *in vitro*. Highly variable spheroid size increases experimental variability if endpoints are quantified, and endpoint analysis often requires sophisticated visual monitoring (33) for quantification. The stereolithographic bioprinting of human SK-N-BE(2) neuroblastoma cells within a photoactivatable methacrylated gelatin matrix that we use here creates a group of identically viable copies with which to conduct sophisticated CAR T cell testing *in vitro*. In this way, high precision and robustness are achieved, which is essential for the comparability of the results within both small and large experimental designs and across experiments. Effector to target ratios also become more difficult to define as models become more complex. The defined structure in our bioprinted 3D model allows precise calculation, while estimation is more difficult for spheroids and almost impossible for *ex vivo* tissue slices and organoids. A closer approximation of physiological organ structure can be achieved in organoids, which can be both organ-specific and patient-specific, and *ex vivo* tissue slices [reviewed in (36)]. Jacob et al. recently described a patient-derived glioblastoma organoid model biobank used to test response to two CAR T cell therapies (37), however, results could only be immunohistochemically examined, increasing evaluation time and complexity. Wallstabe et al. recently present a microphysiological 3D lung and breast cancer model for preclinical CAR T cell evaluation that uses the porcine jejunum as a scaffold (38). An advantage of this model was that fibroblasts and other stromal cells could be included to simulate the immunosuppressive tumor environment, but it is laborious to establish and subject to donor-dependent variance. Higher model complexity reduces comparability among individual experiments, in contrast to our bioprinted 3D tumor models, and reduces accurately quantifiable endpoints that can be used to assess CAR T cell action.

We demonstrated high viability of the bioprinted 3D tumor models shortly after printing and during culture for up to 11 days, in line with results from other groups showing cell viability is preserved in bioprinted 3D tumor models for several weeks (39–44). In addition to maintaining high viability over time, incorporation of other cellular components within the bioprinted 3D tumor model will contribute to recapitulate characteristics of an *in vivo* tumor microenvironment. Langer et al. published a bioprinted 3D tumor model similar to ours, but supplemented with fibroblasts and endothelial cells (45) demonstrating that 3D bioprinting can be used to create complex and heterotypic tumor tissue that incorporate both cancer and stromal cell types. Heinrich et al. and Meng et al. have also used methacrylated gelatin for a glioblastoma cell line model containing macrophages or a metastatic lung cancer cell line model, respectively, albeit using different bioprinting techniques (40, 43). The scientific field developing 3D bioprinting possibilities is expanding with an emphasis to more closely recapitulate certain tumor microenvironment characteristics important to the scientific questions being asked and all exceeding the tumor-nearness of monolayer cultures, but 3D models for preclinical CAR T cell evaluation are still rare. We are currently developing protocols allowing inclusion of endothelial and myeloid-derived suppressor cells to create a more representative microenvironment of human tumors in the bioprinted 3D tumor models to answer questions requiring these tumor components.

L1CAM-specific CAR T cells were able to recognize and infiltrate our bioprinted 3D neuroblastoma models, with microscopic assessment verifying deep L1CAM-specific CAR T cell infiltration. Bead-based quantification of T cell infiltration demonstrated antigen-dependent T cell infiltration, since more CAR T cells, regardless of spacer length, infiltrated the bioprinted 3D neuroblastoma models than untransduced T cells. High numbers of CAR T cells had infiltrated our bioprinted 3D models after only 12 hours, peaking at 24 hours then not further increasing. This observed plateau corresponds with the time window when antigen-dependent proliferation begins and might indicate that T cell proliferation and death balanced the cell numbers, creating the observed plateau. Microscopic assessment predicts a higher infiltration of SS-BB/ζ L1CAM-specific CAR T cells compared to LS-BB/ζ CAR T cells. Tumor suppressive mechanisms that could be preventing CAR T cell proliferation can also not be ruled out. Elucidating possible mechanisms would be feasible using our bioprinted 3D models and would be of special interest to understanding the limits of CAR T cell infiltration and expansion in the solid tumor microenvironment. Ando et al. used a similar tumor cell model, where tumor cells were also embedded in methacrylated gelatin, which showed a modest infiltration of HER2-specific CAR T cells (46). In contrast to our model, where CAR T cell infiltration was already detected after 12 hours, they detected low CAR T cell infiltration after 72 hours. The shape of the bioprinted 3D tumor models, tumor entity used and/or CAR T cell architecture may contribute to differences in T cell infiltration achieved in the different models. The possibility to study CAR T cell infiltration into our bioprinted 3D model allows early and late time point comparisons among selected T cell subtypes *in vitro.*

Assessing antigen expression on tumor cells or activation marker expression on T cells is essential to evaluate novel CAR T cells. We developed a method to preserve protein expression on both tumor and T cells, after obtaining a single-cell suspension. Activation marker expression on L1CAM-CAR T cells with the long spacer was higher than on their counterparts with the short spacer after experiments in either the 2D or 3D models, while both cell types demonstrated comparable cytotoxicity *in vitro*. We have previously shown that *in vivo* function of L1CAM-CAR T cells with a long spacer element was inferior compared to L1CAM-CAR T cells harboring a short spacer in mouse models (19). The more highly activated phenotype of long-spacer L1CAM-CAR T cells observed here *in vitro* might be detrimental *in vivo* where CAR T cells are subjected to repeated antigen encounter leading to activation-induced cell death. So far, we could not predict this phenomenon with our 3D tumor model. This could be due to the relatively low E:T ratio achieved in the 3D coculture system, as only a fraction of T cells introduced to the 3D model actually entered it, resulting in delayed and reduced killing compared to the 2D coculture system. Titrating the number of CAR T cells added to the 3D tumor model to achieve comparable E:T ratios as in 2D coculture experiments are future refinements planned to improve 3D model predictability.

CAR T cells can mediate tumor-specific cytotoxicity, for example by releasing IFNG, which is necessary for complete tumor eradication (47), or by inducing the Fas/FasL axis (48). Unexpectedly, even when CAR T cells were highly activated after coculture with bioprinted 3D tumors, extremely low IFNG release was measured in comparison to 2D cultures. The lower IFNG levels in culture medium from CAR T cell-treated 3D tumors may have been caused by IFNG sequestration in the extracellular matrix of the bioprinted 3D tumors (49). However, the more likely reason for the lower levels is that only a fraction (approximately 10%) of the added CAR T cells infiltrated the 3D tumor model, reducing the amount of T cells able to produce IFNG after encountering the tumor cells, compared to the situation in monolayer co-culture. In line with this finding, L1CAM-specific CAR-mediated cytotoxicity in bioprinted 3D tumors was detected with delayed onset of 5 days and was lower than in 2D cocultures. Calculation of T cell infiltration with quantification beads shifted the effector to target ratio from, the initially added, 5:1 to 1:10 that were actually present within the 3D bioprinted model. This adjusted ratio resolves the lower tumor cell lysis and IFNG release by CAR T cells in the bioprinted 3D tumor model compared to 2D cocultures. Our initially selected 5:1 ratio was chosen only as the initial quantity of T cells to add to cocultures to initiate comparisons between the 2D and 3D models. Corroborating our results, Schnalzger et al. showed that CAR-NK92 cells induced significantly lower tumor cell lysis in 3D compared to 2D models (50). These results in our and other bioprinted 3D models indicate that cytokine release and tumor cell cytotoxicity may be overestimated by 2D coculture testing, and demonstrate that analyses in 3D models are a more effective mirror of real-life obstacles CAR T cells need to bypass in tumors.

We present a 3D tumor model produced in parallelized batch bioprinting production for use in preclinical investigations of CAR T cell effector function and as a potential preselection tool for CAR T cell constructs. CAR T cell infiltration into the bioprinted 3D tumor model proved quantifiable using two different methods, supporting comparisons of the impact of different CAR constructs on T cell infiltration. Single-cell suspensions released from completed experiments retain cell surface proteins and viable cells for quantitative and qualitative functional assessment. This highly reproducible bioprinted 3D human tumor model is a tumor-near *in vitro* environment for CAR T cell preselection based on effector functions prior to *in vivo* studies. Knowing that evaluating tumor infiltration and functionality *in vivo* is essential for CAR T cells developed to treat solid tumors, we do not mean to suggest that our 3D neuroblastoma model can replace CAR T cell evaluation in mouse models, but make *in vitro* testing more stringent so that fewer candidates better adapted to enter solid tumors proceed to *in vivo* testing, thus increasing the speed and reducing the cost (and animal use) of thorough preclinical testing. To best illuminate the particular usefulness of the bioprinted 3D model, we selected a pair of thoroughly preclinically tested CAR constructs and repeated *in vitro* evaluation in direct comparison to the bioprinted 3D model in the experiments presented here. Human neuroblastomas contain a richer tumor tissue environment than our bioprinted 3D model, which while providing the 3D environment lacks specific matrix molecules and cellular components in human tumors. Modeling the human extracellular matrix is, however, a difficult problem also faced in mouse models. Current preclinical NSG mouse models bearing xenografts cannot completely reflect the tissue environment in human tumors due to species-specific discrepancies of chemokine and adhesion molecules resulting in limited trafficking and extravasation of CAR T cells (51, 52). Even patient-derived xenograft mouse models have shown that the tissue environment of the original tumor is rapidly replaced by a murine stroma after a few passages (53). To approach testing of individual extracellular and cellular tumor matrix components (including different patient-derived tumor cell backgrounds, target antigens and tumor entities) *in vitro*, we plan to further adapt this bioprinted 3D tumor model as a part of a future pipeline planned for standard preclinical *in vitro* evaluation of CAR constructs. A gelatin-based ECM in the bioprinted 3D model could present a more tumor-near microenvironment. Following Langer et al., the addition of fibroblasts and endothelial cell layers would benefit this model, especially to investigate the infiltration capacity of CAR T cells into the immune-suppressive environment using human-derived stroma cells (45). These potential adaptations, once standardized, could be applied to evaluate the impact of distinct parameters from tumor matrix components in experimental series. The implementation of additional cell types representing important tumor components with T cell efficacy-influencing properties, such as an immunosuppressive tumor stroma or tumor blood vasculature, will further refine our model to create an *in vitro* tool potentially capable of reducing both the time and animals necessary for preclinical testing in CAR T cell research.

## Data Availability Statement

The original contributions presented in the study are included in the article/**Supplementary Material**. Further inquiries can be directed to the corresponding author.

## Ethics Statement

The studies involving human participants were reviewed and approved by Charité ethics committee approval EA2/262/20. The patients/participants provided their written informed consent to participate in this study.

## Author Contributions

AKü and LK conceived the project. LG performed and analyzed the CAR T cell experiments with the assistance of LA, AK, SS, and AW. TL developed the bioprinted 3D tumor model with assistance of AT. FK was consulted for statistical evaluation. AG and AH-H contributed their help with the immunofluorescence staining. HD, AH, AE and JS gave scientific advices. LG, TL, KAs, KAn, LK, and AKü wrote the manuscript. All authors contributed to the article and approved the submitted version.

## Funding

LG is participant in, and is partly funded by, the “Berlin School of Integrative Oncology”. AH and AKü are participants in the BIH-Charité Clinician-Scientist Program funded by the Charité – Universitätsmedizin Berlin and the Berlin Institute of Health. A research grant (#2017_A51) from the Else Kröner-Fresenius foundation (to AKü) and from Charité 3R| Replace - Reduce - Refine (to AKü) supported this work. AG is supported by the Deutsche José Carreras Leukämie Stiftung (R03/2016). The funders had no role in study design, data collection and analysis, decision to publish or preparation of the manuscript.

## Conflict of Interest

TL, AT and LK were employed by Cellbricks GmbH Berlin.

The remaining authors declare that the research was conducted in the absence of any commercial or financial relationships that could be construed as a potential conflict of interest.
