# Supplementary material for: A Reproducible Bioprinted 3D Tumor Model Serves as a Preselection Tool for CAR T Cell Therapy Optimization
Source: Front Immunol. 2021 Jun 29;12:689697. doi: 10.3389/fimmu.2021.689697 (PMC8276678; doi:10.3389/fimmu.2021.689697)
Supplement: Supplementary file 1 [file DataSheet_1.pdf]

Supplemental Figures

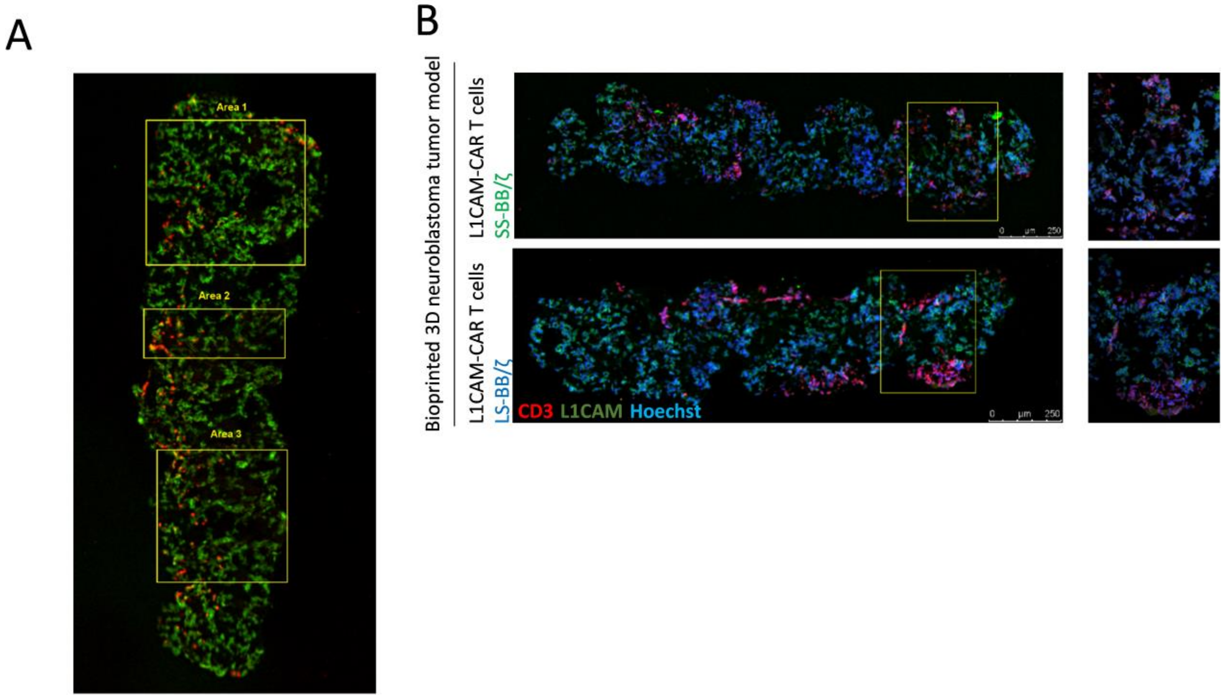

Supplementary Figure 1: CAR T cell infiltration into bioprinted 3D tumor models. A. Representative full image of FFPE tumor model with examplary three sections which were chosen to identify T cell infiltration depth for analysis. **B.** Immunofluorescence staining of Formalin-Fixed Paraffin-Embedded (FFPE) 3D tumor models in co-culture with untransduced T cells and L1CAM-CAR T cells for CD3 (red), L1CAM (green) and Hoechst (blue). Enlarged images of selected regions (yellow) are depicted. Scale bar = 250 µm

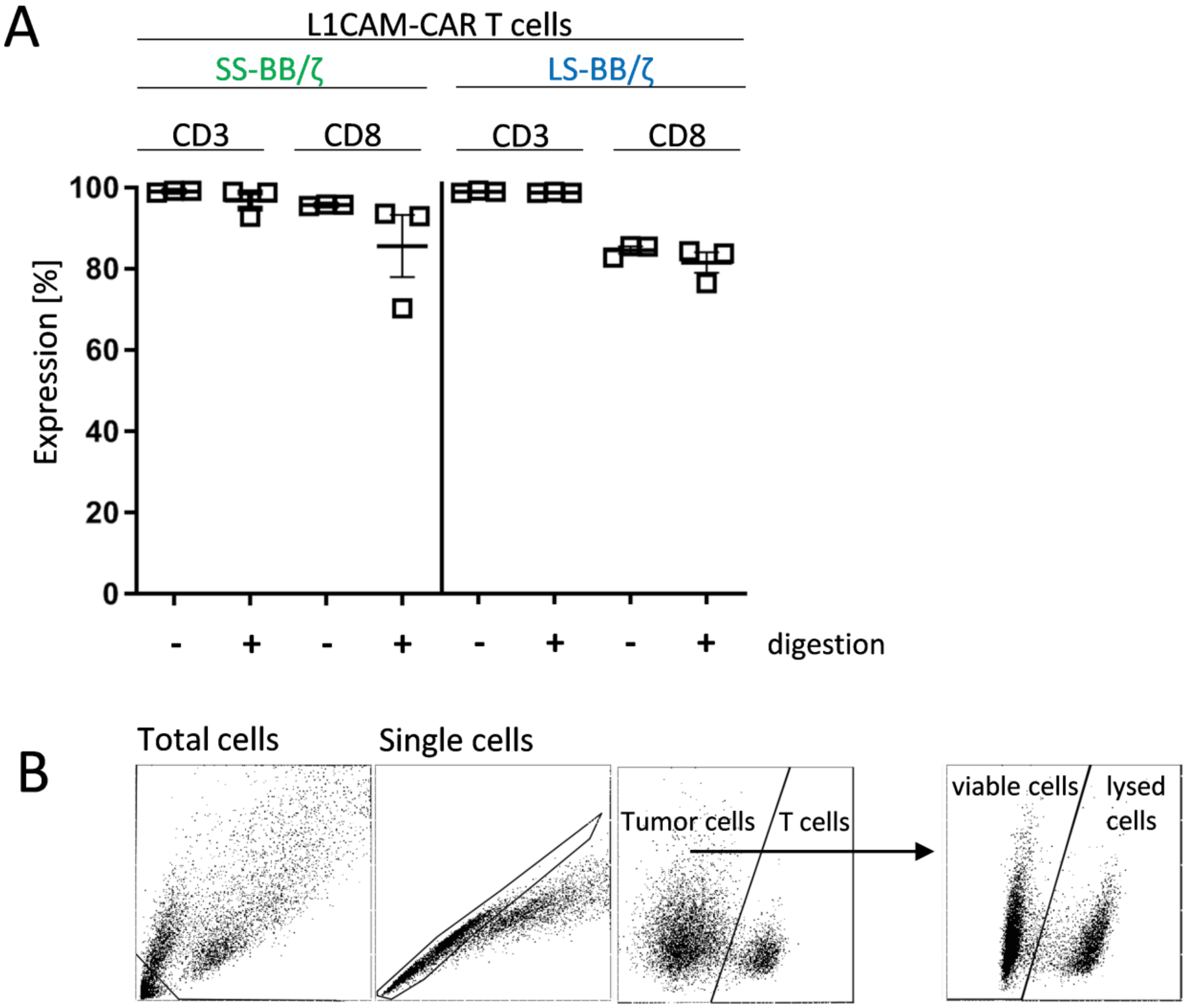

Supplementary Figure 2: Surface expression of L1CAM-specific CAR T cells after enzymatic digestion. A. Flow cytometry analysis for $CD3^+$ and $CD8^+$ surface molecule expression on SS-BB/ ζ and LS-BB/ζ CAR T cells without and with enzymatic digestion are summarized in a scatter plot. Depicted is mean of biological triplicates (2D) or quadruplicate (3D) with error bars representing SD, *, p≤0.5; **, p≤0.01. **B.** Gating strategy applied with FlowJo_V10 after flow cytometry analysis is represented with flow cytometry blots. The gates for total cells (tumor and T cell gate), single cells (single cells) and to differentiate between CD3-negative (tumor cells) and CD3-positive (T cells) cells are applied before analysis of viable cells (live) and dead tumor cells.
